# Oncolytic Viruses for the Treatment of Bladder Cancer: Advances, Challenges, and Prospects

**DOI:** 10.3390/jcm11236997

**Published:** 2022-11-26

**Authors:** Henglong Hu, Qidong Xia, Jia Hu, Shaogang Wang

**Affiliations:** Department of Urology, Tongji Hospital, Tongji Medical College, Huazhong University of Science and Technology, No. 1095 Jiefang Avenue, Wuhan 430030, China

**Keywords:** bladder cancer, oncolytic virus, oncolytic viral therapy, immunotherapy

## Abstract

Bladder cancer is one of the most prevalent cancers. Despite recent advancements in bladder cancer therapy, new strategies are still required for improving patient outcomes, particularly for those who experienced Bacille Calmette–Guerin failure and those with locally advanced or metastatic bladder cancer. Oncolytic viruses are either naturally occurring or purposefully engineered viruses that have the ability to selectively infect and lyse tumor cells while avoiding harming healthy cells. In light of this, oncolytic viruses serve as a novel and promising immunotherapeutic strategy for bladder cancer. A wide diversity of viruses, including adenoviruses, herpes simplex virus, coxsackievirus, Newcastle disease virus, vesicular stomatitis virus, alphavirus, and vaccinia virus, have been studied in many preclinical and clinical studies for their potential as oncolytic agents for bladder cancer. This review aims to provide an overview of the advances in oncolytic viruses for the treatment of bladder cancer and highlights the challenges and research directions for the future.

## 1. Introduction

Bladder cancer (BC) is one of the most prevalent malignancies, with approximately 550,000 new cases every year [1,2]. The transurethral resection of bladder tumor (TURBT) and subsequent intravesical therapy (IVT) are the standard treatments for nonmuscle invasive bladder cancer (NMIBC) [3], while for patients with T2-T4a muscle-invasive bladder cancer (MIBC), radical cystectomy is recommended [4]. In addition, systematic chemotherapy is the first-line therapeutic method for metastatistic cancer [5]. While these therapeutic approaches may provide successful curative options, more treatment methods are still required to further improve the outcomes of BC patients, especially those who suffered Bacille Calmette–Guerin (BCG) failure and those with locally advanced or metastatistic BC.

Over the past ten years, immunotherapeutic approaches for the treatment of BC have gained popularity in both preclinical research and clinical practice [6]. One of the immunotherapy drugs is immune checkpoint inhibitors (ICIs). ICIs have gained great success in the treatment of BC, from patients who are unresponsive to BCG to MIBC patients who require systemic neoadjuvant or adjuvant immunotherapy. In addition, oncolytic viruses (OVs) represent another cutting-edge and promising immunotherapeutic strategy for cancer. Reports of viruses having therapeutic benefits in cancer started appearing at least one century ago [7]. Interest in OVs reached fever pitch in the 1950s, followed by near abandonment in the 1970s and 1980s, and a resurgence of interest in recent years [7]. OVs have significant benefits over other tumor immunotherapies, including precise targeting, few adverse effects, fewer drug resistance, and high killing effectiveness [8]. This review aims to provide an overview of the advances in OVs for the treatment of BC and highlights the challenges and prospects for the future.

## 2. OVs and Their Antitumor Mechanisms in BC

The use of viruses as potential treatments for many diseases has gained more and more attention [9]. OVs are either naturally occurring or purposefully engineered viruses that have the ability to selectively infect and lyse tumor cells while avoiding causing excessive damage to healthy cells [10,11]. Nonpathogenicity, selectively targeting and killing cancer cells, and the ability to be engineered to express tumor-killing substances are common characteristics of OVs [12]. BC is a good candidate for oncolytic immunotherapy. These are the causes: (1) Intravesical therapy for BC using BCG or other drugs is well established in clinical practice; (2) through intravesical instillation, the BC can be exposed to high virus titers; and (3) the surface area for topical application is increased by the papillary structure of BC [13,14].

**Figure 1:**
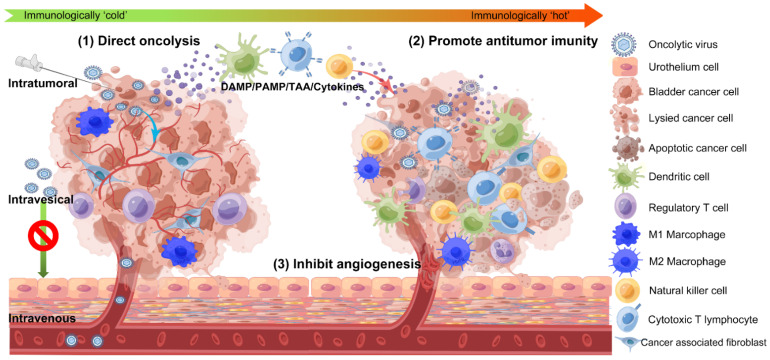
**Proposed antitumor mechanisms of oncolytic viruses in bladder cancer.** The oncolytic viruses (OVs) can be administered intravenously, intravesically, and intratumorally. OVs selectively infect bladder cancer cells while avoiding harming healthy cells. After the infection, OVs replicate in bladder cancer cells and finally lyse the malignant cells. The infection and direct oncolysis result in the release of cell-derived damage-associated molecular patterns (DAMPs), viral pathogen-associated molecular patterns (PAMPs), soluble tumor-associated antigens (TAAs), and various cytokines. These molecules recruit and activate antigen-presenting cells such as dendritic cells (DCs), natural killer (NK) cells, and other immune cells to the infection site. DCs take up soluble tumor antigens and then activate adaptive T cell responses against the tumor at regional lymph nodes. NK cells and cytotoxic T lymphocytes can recognize and kill tumor cells. Finally, OVs break down the immune suppressive tumor microenvironment that consists of immunosuppressive cells and a dense stroma to establish an immunologically “hot” tumor microenvironment. This remodeling promotes the immune system to recognize and kill primary, metastatic, or recurrent malignant cells. In addition, many OVs can effectively suppress intratumor angiogenesis and reduce the supply of nutrients and oxygen to the tumor by direct lysing the vascular endothelial cells, instigating the formation of microthrombosis, and expressing anti-angiogenesis viral proteins.

In preclinical and clinical studies, several OVs have been studied for the treatment of BC; however, the precise anticancer mechanisms of OV in BC are still not well understood and may differ from virus to virus. As shown in Figure 1, the main antitumor mechanisms include [15]:

### 2.1. Direct Oncolysis

Malignant cells are particularly vulnerable to OVs infection due to tumor-driver mutations in cancer cells and particular cytokines that these cells produce [16]. For example, numerous tumor cells sustained preferential virus multiplication, which was likely brought on by a deficit in type I interferon’s antiviral signaling [17,18]. In addition, some OVs are molecularly engineered to infect cancer cells specifically [11]. Once infection takes hold, OVs will take control of the tumor cell’s production line for nucleic acids and proteins, preventing the cancer cells from producing enough nucleic acids and proteins to meet their growth requirements and destroying their normal physiological processes [15]. The viruses cause alterations in cell function and ultimately kill and lyse the cancer cells by damaging organelles [19,20,21,22]. Then more OVs are released and spread to nearby cancer cells. OVs may infect healthy cells, but they are unable to proliferate there because these cells have normal antiviral capability and response [11].

### 2.2. Promoting Antitumor Immunity

The tumor microenvironment (TME) of advanced malignancies is “cold” as lacking anti-tumor immune activity [23]. OVs can directly lyse malignant cells and lead to the release of cell-derived damage-associated molecular patterns (DAMPs), viral pathogen-associated molecular patterns (PAMPs), and soluble tumor-associated antigens (TAAs). These molecules recruit and activate antigen-presenting cells such as dendritic cells (DCs), natural killer (NK) cells, and other immune cells to the infection site. DCs take up soluble tumor antigens and then activate adaptive T cell responses against the tumor at regional lymph nodes. Additionally, enhanced antigen processing and presentation factors and tumor-specific CD8+ T lymphocytes recruitment are brought about by the viral-mediated production of chemokines and type I interferons. These cytotoxic T lymphocytes can recognize and kill both primary and metastatic tumor cells. Interferons’ counterregulatory effects can also increase the production of immunological checkpoint molecules by tumor cells, such as galectin 9 and cytotoxic T lymphocyte-associated antigen 4 (CTLA-4), as well as programmed cell death 1 ligand 1 (PD-L1) [24]. The immune suppressive TME is finally broken down by OVs to produce an immunologically “hot” TME that promotes the eradication of primary, metastatic, or recurrent tumor cells [25,26,27].

### 2.3. Inhibition of Intratumor Angiogenesis

Angiogenesis assumes a significant part in tumor growth and development [28]. By directly lysing the vascular endothelial cells, causing microthrombosis and producing anti-angiogenesis viral proteins, some OVs can successfully suppress intratumor angiogenesis and reduce the supply of nutrients and oxygen to cancer cells, thus preventing the proliferation of tumor cells [29,30,31].

## 3. OVs for BC

Initially, wild-type viruses were used in oncolytic virotherapy (OVT). To improve the effectiveness of treatment, second-generation OVs were built on genetically engineered viruses. Third-generation OVs are “armed viruses” that have been cloned with immune stimulatory or toxic genes such as granulocyte macrophage colony-stimulating factor (GM-CSF) and interleukin-2 to accelerate resistant antitumor immunity and increase tumor destruction [32,33]. A wide diversity of viruses have been investigated for their potential to act as oncolytic agents for BC, including adenoviruses, herpes simplex virus (HSV), Coxsackievirus, alphavirus, vaccinia virus, Newcastle disease virus (NDV), and vesicular stomatitis virus (VSV), et al. [34,35,36,37,38,39,40,41,42,43,44,45,46,47,48,49,50,51,52,53,54,55,56,57,58,59,60,61,62,63,64,65,66]. Table 1 listed the OVs in preclinical studies.

### 3.1. Adenovirus

Adenovirus is the most explored and studied OVs in BC. As a double-stranded DNA virus, it has an icosahedral capsid and infects the cell through Coxsackie and adenovirus receptors [65]. To increase antitumor effectiveness and cancer cell selectivity, many genetically modified adenoviruses have been created. In 2002, Zhang et al. published their pioneering work in this area [60]. They engineered Uroplakin II (UPII) promoter into adenovirus type 5 to create an attenuated replication-competent adenovirus variant termed CG8840. In contrast to normal cells, CG8840 was highly selective (10,000:1) and capable of efficiently replicating in and eliminating BC cells. Additionally, the injection of CG8840 intravenously and intratumorally to RT4 human BC xenografts significantly slowed the growth of the tumors [60]. Then, Ramesh et al. constructed CG0070, a serotype 5 adenovirus that contains the cDNA for human GM-CSF [51]. GM-CSF is well known for being a strong inducer of specific and persistent anticancer immunity. Several BC models were used in in vitro and in vivo experiments and confirmed the GM-CSF production, cytotoxicity, selective replication, and antitumor effectiveness of CG0070 [51]. Results from preclinical studies led researchers to assess the safety, pharmacokinetics, and anticancer activity of CG0070 in 35 patients with NMIBC. According to the phase I trial, intravesical therapy is safe and generated considerable anticancer activity [67]. Additionally, the overall CR rate for BCG-unresponsive NMIBC patients is 47% according to the phase II study’s 6-month interim results. The CR rate for patients with pure CIS was 58%, and it was 50% in BC patients whose tumors incorporating CIS [68]. In an orthotopic model, Wang et al. discovered that the direct intravesical instillation of AxdAdB-3, a oncolytic adenovirus with the deletion of E1B-55KD and mutant E1A, dramatically slowed the growth of the bladder tumor [58]. Lichtenegger et al. demonstrated that the intratumoral delivery of XVir-N-31, a YB-1-selective adenovirus, significantly inhibited tumor development and caused higher immunogenic cell death (ICD) in BC cells than wild-type adenovirus [44]. Furthermore, Lu and his colleagues evaluated the teratogenic toxicity of BC-specific adenovirus Ad-PSCAE-UPII- E1A-AR on mice and determined that it was safe in pregnant mice and had no discernible effects on the development of F1 mice [47]. There are numerous other OVs that have been studied preclinically in BC, for example, Ad-MK-E1a [55], Ad.9OC [59], Ad5F35/MKp-E1 [36], Ad/PSCAE/UPII/E1A [62,63,69], RGD-hTERT-TRAIL [64], AdLCY [46]. Most of the results are encouraging.

### 3.2. Herpesvirus

HSV is a double-stranded DNA virus with enclosed virions that has the ability to remain latent in host cell neurons [70,71]. HSV-1 is quite prevalent; about 67% of people worldwide have ever been exposed to it [72]. Many different oncolytic HSVs (T-VEC, G207, HSV1716, NV1020, HF10, et al.) have been designed and developed to treat a variety of malignant tumors, including BC [71,73,74]. However, the main issue with applying HSV-1 in clinical practice is that it is neurotropic, which could increase the risk of neurovirulent side effects when used in clinical settings. To decrease this risk, recombinant HSV-1s were developed by deleting the neurovirulent genes such as the diploid 134.5 genes and thymidine kinase (TK). G207 is a genetically engineered OV based on wild-type HSV-1. The deletion of γ134.5, which results in greatly decreased neurovirulence, is one of G207’s distinguishing characteristics [75]. NV1020 is another HSV-1 mutant with a loss of 700-bp in the TK gene [74,75]. All four human BC cell lines and MBT-2 cells were susceptible to infection, internal replication, and lysing by both viruses. In vivo research showed that these viruses were efficient at reducing tumor burden in syngeneic C3h/Hej mice with a single intravesical instillation and even more effective with multiple instillations [74]. In 2005, the effectiveness of a HSV-1 mutant HF10 for regulating the proliferation of human and mouse BC cells was examined by Kohno et al. in vitro and in vivo [42]. They discovered that HF10 replicated effectively in MBT-2 and T24 BC cells and caused significant cell lysis. In mice with disseminated peritoneal and BC models, the treatment of HF10 markedly increased survival rates and lengthened survival durations [42]. There are also some similar HSV-1 mutants such as NV1066. According to Mullerad et al., NV1066 synergistically increased MMC’s cytotoxicity for BC cell lines (KU19-19 and SKUB) [48]. However, these deletions unavoidably diminish the oncolytic effectiveness and replication efficiency of HSV-1 [76]. Therefore, Zhang et al. designed a recombinant virus and tried to regulate the expression of genes essential for replication with endogenous microR143 and microR124. They found that the miR143/124-regulated HSV-1 could restrict viral replication in neurons and normal bladder cells while killing BC cells with high potency. Thus, it is possible to maintain the full viral genome for the greatest oncolytic potency while maintaining the highest level of safety by translationally regulating the expression of essential viral genes [65].

Pseudorabies virus (PrV), a neurotropic herpesvirus, infects a variety of hosts but is nonpathogenic for humans [77,78]. Shiau et al. generated YP2 virus, a Glycoprotein E/TK-defective PrV mutant carrying both Glycoproteins D and HSV-1 TK genes under the transcriptional control of the HER-2/neu promoter [54]. In MIBC, there has been evidence of HER-2/neu overexpression, which is associated with worse clinical outcomes and increased metastases [79,80]. It enhances cancer cell survival, invasion, and angiogenesis, leading to increased cancer metastases and resistance to various cancer therapies [80]. Researchers found that YP2 selectively lysed HER-2/neu-overexpressing mouse and human BC cells. In addition, YP2 significantly inhibited the growth of the MBT-2 bladder tumor in mice [54].

Recently, Joo et al. constructed an oncolytic virus from HSV-2 termed FusOn-H2 that targets cancer cells selectively by activating the signaling pathway of Ras [40]. In an orthotopic murine BC mode, they assessed the anticancer activity of FusOn-H2. They found that in the majority of the animals, two moderately dosed intravesical instillations of the virus completely eliminated the tumors. Additionally, FusOn-H2 triggered a potent systemic immune response to the native tumor antigens created by tumor cells. They also compared FusOn-H2 with an oncolytic HSV-1 (Baco-1) and discovered that FusOn-H2 had considerably higher anticancer efficacy [40]. According to their findings, FusOn-H2 may act as an effective oncolytic drug for orthotopic BC.

### 3.3. Coxsackievirus

A naturally occurring common cold RNA virus known as Coxsackievirus A21 (CVA21) has demonstrated specific oncolytic activity in many tumors [81]. In a panel of human BC cell lines, Pandha et al. studied CVA21-induced cytotoxicity and discovered a variety of sensitivities that were largely correlated with the expression of the viral receptor ICAM-1 [34]. They also discovered the expression of the ICD determinant calreticulin and the release of HMGB-1 in CVA21-treated BC cell lines, which indicated that CVA21 could induce immunogenic apoptosis [34]. The results of this study suggest that CVA21 has the potential to become a new oncolytic immunotherapy drug for NMIBC. In addition, the researchers found that low doses of MMC could enhance CVA21′s oncolysis and replication through increasing ICAM-1 expression [82].

Based on these findings, a phase I/II trial (CANON, NCT02316171) was conducted to investigate the therapeutic potential of CVA21 (CAVATAK) for NMIBC. This trial included 15 NMIBC patients who were candidates for TURBT and evaluated the feasibility, safety, and biological effects of escalating intravesical doses of CAVATAK, either alone or combined with MMC. The production of tumor inflammation and bleeding after intravesical installations of CAVATAK served as clinical evidence of the drug’s activity. CAVATAK induced significant inflammatory alterations within NMIBC tissue whether it was used alone or in combination with MMC. One patient had a complete resolution of the tumor. Regardless of whether a patient was getting viral or combination therapy, no severe toxicities were reported [83].

### 3.4. Vesicular Stomatitis Virus

VSV, a negative-sense RNA virus with an envelope, can selectively replicate in IFN-resistant cancer cells [17,84]. IFN resistance favors tumor growth over normal cells but impairs the cancer’s ability to fight viruses [85,86]. This vulnerability of tumor cells is present in many malignancies [87]. A study assessed 57 cancer cell lines and found that 47 of them were sensitive to VSV oncolysis [88].

Hadaschik et al. treated four human BC cell lines (KU-7, UM-UC3, MGH-U3, and RT4) with either a mutant d51M variant (AV3) or wild-type VSV [37]. They discovered that the IFN-nonresponsive and more aggressive BC cell lines UM-UC3 and KU-7 were more frequently destroyed by AV3 and wild-type VSV, whereas IFN-responsive RT4 and MGH-U3 BC cells were less vulnerable. Intravesically administering type VSV and AV3 both dramatically reduced the growth of the KU-7 tumor in mice by 98% (wild-type) and 90% (AV3). These discoveries provide preliminary evidence supporting the intravesical use of VSV in NMIBC patients, particularly those with IFN resistance [35]. Furthermore, they found that type I interferon receptor down-regulation made BC cells more susceptible to VSV-induced cell death [66].

Recently, Rangsitratkul et al. armed VSVd51 with GM-CSF [52], treated human and mouse BC cells or spheroids with VSVd51-m/hGM-CSF, and observed the enhanced release of immunogenic factors and anger signals. Additionally, the intravenous administration of the OV increased survival and decreased tumor volume in MB49 BC-bearing C57Bl/6 mice and promoted the activation of bladder-infiltrating and peripheral effector immune cells [52]. These results suggest that the engineered VSVd51-hGM-CSF may be a promising OV for BC.

### 3.5. Alphavirus

Oncolytic alphaviruses have seen investigated to treat different types of malignancies such as brain cancers, leukemia, melanomas, lymphomas, and BC [89]. A Getah-like alphavirus strain with positive single-strand RNA called M1 was discovered in China’s Hainan Province [90]. The M1 virus has the ability to specifically reproduce in cancer cells, which allows it to eliminate them without seriously affecting healthy organs [91]. The zinc-finger antiviral protein (ZAP) gene regulates M1’s replication, which has a powerful and selective anticancer effect. According to a study of cancer tissue banks, 61% of BC tissue has low levels of ZAP, which suggests that M1 has a wide range of potential applications [19]. M1 caused endoplasmic reticulum stress, which led to apoptosis [19] Additionally, M1 can make cancer cells more sensitive to them when the cyclic adenosine monophosphate pathway is activated [92].

Orthotopic MIBC mice given M1 treatment had significantly slower tumor growth and longer survival times [39]. M1 has more potent antitumor effects than the first-line chemotherapeutic drug cisplatin. Decreased Ki-67 signals and enhanced cleaved-caspase-3 signal, which are indicators of cell proliferation and death, respectively, were seen in treated tumors [39]. This indicates that M1 is a novel oncolytic agent for MIBC. The M1 virus is susceptible to the antiviral effects of coiled-coil-domain-containing 6. And knocking down it increased M1’s oncolytic effects through endoplasmic reticulum stress-mediated apoptosis [45].

### 3.6. Newcastle Disease Virus

The NDV genome is a nonsegmented, single-stranded, negative-sense RNA, and it is a pleomorphic enveloped virus with a diameter of 200–300 nm [93]. Despite the possibility of modest transient flu-like symptoms or conjunctivitis, NDV is typically not harmful to people [32]. Numerous human cancers with pathogenic (Ulster, PV701, and MTH-68/H) and nonpathogenic (73-T, LaSota, HUJ, and Hitchner-B1) viral strains have shown that NDV has oncolytic potential [32]. As early as 1992, the cytolytic activity 73-T was determined on six human tumor cell lines including BC cells (HCV29T). The researchers found that the intratumoral injection of 73-T caused full tumor regression in mice and that the drug selectively and effectively lysed BC cells [53]. Infected human and mouse cells with NDV cause ICD, the activation of innate immune pathways, and the elevation of major histocompatibility complex and PD-L1, according to a recent discovery by Anton Oseledchyk [49]. Intratumoral therapy with NDV enhanced immune infiltration and effected a change from an inhibitory to an effector T cell phenotype in both treated and untreated BC tumors [49]. Improvements in local and distant tumor control and overall survival have been seen when intratumoral NDV was combined with systemic programmed cell death protein 1 (PD-1) or CTLA-4 inhibition [49]. These results support more clinical studies combining intratumoral NDV treatment with systemic immunomodulatory drugs.

### 3.7. Reovirus

Reovirus is a nonenveloped, double-stranded (ds) RNA virus with 2 concentric icosahedral protein capsids, measuring around 85 nm in diameter [94]. Although reovirus can be found in the human respiratory and gastrointestinal tracts, it is not known to be harmful. Reovirus’s oncolytic abilities seem to be somewhat reliant on Ras signaling. Ras transformation also influences many phases of the viral life cycle, which helps to increase reovirus oncolysis [95]. Reovirus was shown to have the ability to destroy rat AY-27 BC cell lines and human BC cell lines (RT-112 and MGH-U3) in a preclinical investigation by Kilani et al. [41]. Using an orthotopic bladder tumor model, Hanel et al. reported the first intravesical oncolytic reovirus for the treatment of NMIBC [38]. When compared with the BCG group, the reovirus group’s side effects were fewer. Reovirus treatment significantly increased tumor-free survival compared with BCG or standard saline treatment in animals [38].

### 3.8. Vaccinia Virus

The double-stranded DNA virus vaccinia virus (VV), which has a genomic length of roughly 190 kb, offers a number of features that make it a promising OVT agent [96,97,98]: (1) VV has been crucial to the effectiveness of the smallpox vaccine. VV has a long history of usage in people with success, which implies that it is a secure oncolytic drug. (2) VV has a vast genome; a significant amount of foreign DNA can be inserted without affecting the virus’s ability to reproduce. VV stays in the cell cytoplasm throughout the infectious cycle, in contrast to other groups of DNA viruses [97,98]. VV has been studied in a variety of human malignancies including BC [97,98,99]. The F4L gene, which codes for the virus’s homolog of the ribonucleotide reductase’s cell-cycle-regulated small subunit, is a crucial part of VV virulence, and viral strains lacking the F4L gene exhibit in vivo attenuation [50]. By muting F4L, Potts et al. created a new oncolytic VV that selectively replicates in BCG-resistant BC cells (AY-27) and xenografted human RT112-luc orthotopic BC mice, significantly inhibiting tumor growth without producing any apparent side effects [50]. Their research offers patients with BC who are resistant to BCG a potentially effective treatment.

## 4. Clinical Trials of OVs for BC

So far, four OV-based drugs have been approved for cancer treatment globally. Rigvir, a picornavirus, was the first OV approved in Latvia to treat melanoma in 2004 [100]. In November 2005, Oncorine, an engineered adenovirus with the designation H101, received approval to treat head and neck cancer in China [101]. Ten years later, in 2015, Talimogene Laherparepvec (T-VEC), a modified HSV-1, was authorized for use in the USA and Europe to treat nonresectable metastatic melanoma [102,103]. Last year, another engineered HSV named DELYTACT received approval in Japan for the treatment of malignant glioma [104].

However, no OV has been approved to treat BC around the world, although some OVs are in clinical research. As early as 2001, Gomella et al. first explored the safety of direct delivery of live virus into the human bladder. They demonstrated that VV can be administered safely into the bladder with the recruitment of lymphocytes and the induction of a brisk local inflammatory response. We searched the Chinese Clinical Trial Registry and ClinicalTrials.gov websites and found that the number of clinical trials focusing on OVs in BC is relatively small. The registered clinical trials, either ongoing or completed, are shown in Table 2. Most of them are phase I clinical trials, and only one trial is in phase III. Unlike other tumors that have been studied primarily in advanced stages, OVs are studied in NMIBC, MIBC, and metastatic tumors. The CANON study (NCT02316171) determined the safety, feasibility, and biological effects of CVA21 (CAVATAK) in NMIBC patients before TURBT surgery, and the results have been presented above and in Table 2 [83]. The main OV against BCG-unresponsive NMIBC is CG0070. The promising results of phases I and II of CG0070, as described above, have led to CG0070 becoming the only OV that have been studied in the phase III clinical trial for BC [67,68]. Another OV developed for BCG-unresponsive NMIBC is OH2 [105]. It belongs to the genetically modified HSV-2 strain HG53 and can selectively replicate itself in tumors [105,106]. Meanwhile, the transfer of GM-CSF may induce a stronger antitumor immune response [106]. Two other OVs are in phase I clinical trials in patients undergoing radical cystectomy, Enadenotucirev and MV-NIS. Enadenotucirev is an adenovirus, and the phase I trial showed that following the intravenous infusion, the virus could be detected in the majority of tumor samples, but little or no virus activity had been shown in normal tissue [107]. Additionally, in 80% of the studied tumor samples, the viral delivery resulted in the substantial local infiltration of CD8+ cells [107]. MV-NIS, a measles virus, is being tested as a neoadjuvant immunotherapy agent for high-grade BCG-refractory NMIBC or MIBC undergoing radical cystectomy [108]. Several other viruses are currently underway for patients with advanced BC, including Coxsackievirus, adenovirus, HSV, and vaccinia virus (Table 2). Of these, the trial of OH2 for advanced BC is conducted at our center and is currently recruiting patients [109].

## 5. Combination Therapy

As previously mentioned, OVs possess multiple anticancer mechanisms. It has been hypothesized that using OVT in conjunction with other anticancer treatments including chemotherapy, radiotherapy, and other immunotherapies may increase therapeutic efficacy [110]. In fact, over the past few decades, a range of combination strategies with OVs have been explored in a variety of malignancies including BC [15,110,111].

### 5.1. OVT in Combination with Chemotherapy

Chemotherapy plays an important role in the treatment of NMIBC and MIBC [112]. Actively proliferating cancer cells in the S/G2/M phase are sensitive to chemotherapy, while quiescent cancer cells in G0/G1 phase such as cancer stem cells are refractory to it. Dormant cancer cells can be promoted by OVs to cycle into the early S-phase and become vulnerable to chemotherapy [113]. Additionally, combining chemotherapy with OVT increases apoptosis [114,115].

Zhao et al. discovered that using ONYX-015 and MMC together significantly increased the cytotoxic effect against T-24 cells compared with using either drug alone [61]. Additionally, compared with the sole use of ONYX-015 in vivo, the combined therapy significantly inhibited tumor growth. Following the combined therapy and alone use of ONYX-105, it was possible to see clear signs of T-24 cell apoptosis [61]. Human UPII promoter and prostate stem cell antigen enhancer (PSCAE)-regulated oncolytic adenovirus Ad/PSCAE/UPII/E1A, which carries the E1A gene, has the ability to selectively destroy bladder tumor cells. According to Wang et al., cis-platin and Ad/PSCAE/UPII/E1A treatment dramatically reduced BC cell proliferation and increased apoptosis over single-drug treatment [62]. Furthermore, there was a strong correlation between the elevated Fas expression and the enhanced anticancer effects of Ad/PSCAE/UPII/E1A and cisplatin [62]. Along with MMC, Li et al. also identified another recombinant adenovirus (Ad5-UPII-E1A) that can increase the sensitization of BC cells to hydroxycamptothecin and the drug’s ability to cause apoptosis [43].

This synergistic effect was further validated in oncolytic HSV by Mullerad et al. They used MMC and NV1066, an oncolytic HSV-1 with the deletion of viral genes ICP0 and ICP4, to treat the BC cell lines KU19-19 and SKUB. The combination treatment showed a synergistic impact, enabling dosage reductions for the treatment of BC cell (SKUB and KU19-19) while reaching an estimated 90% cell death [48]. Furthermore, similar results had been discovered in oncolytic Coxsackie A21 virus and MMC [34,35,82].

### 5.2. OVT in Combination with Radiotherapy

Radiotherapy can play an important role in the treatment of BC, not only as a palliative treatment but also as part of a bladder preservation strategy for localized MIBC [116]. Many studies demonstrated that OVT in combination with radiotherapy has a synergistic antitumor effect. Some explanations have been put forth despite the fact that the underlying mechanism of this synergistic antitumor effect is not entirely apparent. OVs may increase the radiosensitivity of tumor cells, increasing their sensitivity to radiation cytotoxicity. Furthermore, radiation may accelerate viral uptake and replication, leading to cell death [117]. Zhang et al. tried to clarify the synergistic effects of Ad-PSCAE-UPII-E1A and radiotherapy in BC cells [63]. Compared with the single treatment, the combination therapy improved antitumor efficacy. BC cells were halted in the G1 or S phase as a result of OVs and radiation-induced cell death. The OV and radiation-treated BC cells showed signs of autophagic vacuoles. After exposure to radiation, the hexon proteins of OVs increased, whereas the proteins related to DNA break repair decreased [63].

### 5.3. OVT in Combination with ICIs

Immune checkpoint blockade (ICB) is a process in which the ICIs attach to immune checkpoint molecules such as PD-1, PD-L1, or CTLA-4 to prevent immunological inhibitory signals. Recently, ICIs have been used in BCG-unresponsive, neoadjuvant, and adjuvant settings to improve BC treatment outcomes [118,119]. However, some individuals do not benefit from ICIs [120]. Since OVs can break down the immunosuppressive TME, they represent intriguing candidates that synergize with ICIs to improve ICB effectiveness [121,122]. Infection with OVs stimulates an immune response against malignant cells, enhancing the effectiveness of ICIs [123,124]. According to the findings of Oseledchyk et al., NDV infection with BC cells led to ICD, the activation of innate immunity pathways, the elevation of the major histocompatibility complex and PD-L1, and innate immune pathway activation [49]. When combined with intratumoral NDV, systemic ICIs improved both local and distant tumor suppression and overall survival [49]. Clinical trials combining OVs with systemic ICIs are encouraged by these findings. According to KEYNOTE-200 (NCT02043665), in advanced BC patients, intravenously administered oncolytic virus CVA21 in conjunction with pembrolizumab was usually well tolerated. Additionally, among BC patients who have never used immune checkpoints, the ORR is 31% (8/26, 3CR + 5PR) [111]. The CORE-001 study (NCT04387461) aims to evaluate the efficacy of intravenous administration of pembrolizumab and intravesical administration of CG0070 in NMIBC patients who are resistant to BCG treatment. This study uses n-dodecyl-B-D-maltoside as a transduction-enhancing agent and now is recruiting patients.

### 5.4. OVT in Combination with Adoptive Cell Therapy

The cancer treatment known as adoptive cell therapy (ACT) has made tremendous strides in recent years. Chimeric antigen receptor T cells (CAR-T), tumor-infiltrating lymphocytes (TILs), and T-cell receptor (TCR) transgenic T cells are the three main ACT treatment techniques [125]. In CAR-T therapy, T cells are equipped with chimeric antigen receptors, which can recognize and eliminate tumor cells [126]. Although several clinical trials for BC patients are using CAR-T technology, CAR-T cell research for the treatment of BC is still in its infancy [127]. In addition, applying CAR-T cells to solid malignancies such as BC has two major challenges. One is the immunosuppressive TME, which inhibits CAR-T cell activity by overexpressing surface inhibitory chemicals and attracting immune suppressor cells, and the other is the lack of TAAs, which are necessary for powerful T cell responses [128]. As OVs can break down the immune suppressive TME and increase the expression of TAA, CAR-T and OVs may have synergetic effects [129]. In 16% of bladder cancers, HER-2 was found to be changed [130]. One trial NCT03740256 is exploring the safety and efficacy of using HER-2-specific CAR-T cells and CAdVEC, an oncolytic adenovirus in HER-2-positive solid tumors including BC. We look forward to the early conclusions of this study.

## 6. Challenges and Prospects

During the last two decades, advancements in genetic engineering and oncology have facilitated the rapid progression of OVs in BC. However, no OV has been approved for the treatment of BC, and only one trial reached phase III. Therefore, searching for and constructing more specific and effective OVs armed with specific receptors, tumor extracellular matrix-degrading enzymes, cytokines, chemokines, costimulatory molecules, and antiangiogenic molecules to improve antitumor effects for BC remains a relentless pursuit [131]. In addition, we believe that research should be also strengthened in the following areas.

### 6.1. Improving the Delivery Efficiency

Drug delivery is the first important step of OVT. Due to the anatomical characteristics of the bladder, intravesical therapy is easy to perform, and OVs may infect bladder cancer cells through membrane receptors [14,23]. However, the distinctive structure of the urothelium together with a negatively charged glycosaminoglycan (GAG) layer imposes barriers to intravesical drugs and may limit OVs’ infection efficacy [14]. Therefore, opening the bladder permeability barrier is of great significance for effective intravesical instillation to treat bladder cancer [132]. Some pretreatment agents have been developed to enhance OVs infection of BC, and more are strategies under research [132,133]. Intravesical therapy facilitates the use of OVs in NMIBC, but it does not apply to metastatic disease. The OVs’ concentration can be precisely controlled by intratumoral injection; however, lesions might not be possible to achieve, and the intricacy of intratumoral injection might prevent repeated dosing [134]. The intravenous injection, which theoretically eliminates practical issues, seems to be ideal for targeting the primary tumor, systemic metastases, and micrometastases. However, due to dilution of the virus, antibody neutralization, and untargeted infection, there is a chance that the virus will not be able to reach the tumor location efficiently [54]. Additionally, even if the OVs reach the lesion directly, the extracellular matrix in the tumor may prevent the distribution of the OVs, decreasing their effectiveness. Some methods such as the co-administration of extracellular matrix-degrading enzymes including relaxin, matrix metalloproteinase−1, −8, −9, chondroitinase, and hyaluronidase with OVT, or the induction of their gene expression, can increase the spread of OVs and improve OVT efficiency [135]. Mesenchymal stem cells, monocytes, and T lymphocytes have also been investigated as carrier cells that can be loaded with the OVs ex vivo and then injected intravenously. In animal models, carrier cells can successfully protect OVs from antibody-mediated neutralization and nonspecific uptake [136]. How to improve the delivery strategy and efficiency of oncolytic drugs according to the characteristics of OVs and tumors remains to be explored in future studies.

### 6.2. Combination Therapy

It has been widely established that different tumor treatments may have different specific mechanisms and that combination therapies may act synergistically. As previously mentioned, the same applies to OVs in combination with other therapies, and more effort should be devoted to this research aspect than just the combination therapy in BC we described above. Some more novel combined treatments can be explored such as photodynamic therapy (PDT) and OVT. PDT is a nontoxic photosensitizer that employs safe visible light to stimulate the production of reactive oxygen species in tumors, which then cause cytotoxicity in tumor cells [137]. PDT has been used as a novel strategy for NMIBC, especially BCG-unresponsive NMIBC [138,139]. A KillerRed-armed oncolytic adenovirus with telomerase-specific replication competence, termed OBP-301, was created by Takehara et al. [140]. This OV could express KillerRed protein, which produces reactive oxygen species (ROS) when exposed to a green laser or fluorescent light. OBP-301 exhibits higher cancer selectivity than standard photosensitizers and only causes sustained tumor accumulation of the KillerRed protein in infected cancer cells [140]. In mice xenografts, OBP-301 with KillerRed and PDT had higher efficacy against colon cancer lymph node metastasis and orthotopic melanoma [141]. Similar approaches can be tested and further developed in the treatment of BC.

### 6.3. Tumor Imaging

Cystoscopy and surgical resection is still the most used diagnosis and treatment method for BC. Real-time intraoperative fluorescence guidance is ideal for the recognition and precise surgical resection of the tumor. Currently, both indocyanine green and methylene blue have been approved for fluorescence-guided surgery. However, these agents are not specific to malignant tumors. Generating OVs that are specific to tumors and express fluorescent protein can be useful for bladder diagnosis and resection [142]. Furthermore, with the support of genetic engineering technology, real-time imaging can also have the potential to visualize and quantify therapeutic efficacy in vivo [143].

## 7. Conclusions

A variety of OVs has shown promising benefits in the field of BC therapy that are primarily attributed to their ability to induce oncolysis and immunomodulation. A combination of OVs and other antitumor strategies may have synergic effects. Research on OVs for BC is still far from adequate. Constructing more specific and effective OVs, improving OVs delivery efficacy, and combining OVs with other antitumor therapy and OVs-based tumor imaging for BC are attractive research directions

## Figures and Tables

**Table 1:** Preclinical studies on oncolytic viruses for Bladder cancer.

Parent Virus	Oncolytic Viruses	Virus Description	Reference
Adenovirus	CG8840	An adenovirus variant engineered with a UII promoter that can drive E1A and E1B expression	[60]
	Ad5-d55K; Ad5-d24	Serotype-5 adenoviruses that have been weakened to selectively replicate in p53-deficient (Ad5-d55K) cells or retinoblastoma pRb (Ad5-d24) cells	[56]
	ONYX-015	Adenovirus that has had its E1B 55-kDa gene removed and has been modified to replicate only in and lyse the cancer cells lacking p53	[61]
	AxdAdB-3	A double-restricted oncolytic virus that carries an E1A mutation and an E1B-55KD deletion	[58]
	CG0070	Adenoviruses armed with GM-CSF	[51]
	Ad-MK-E1a	A conditionally replicating and MK promoter-regulated adenovirus	[55]
	Ad.9OC	Nine copies of Oct-3/4 response element-derived adenovirus with the E1B-55 kDa deletion	[59]
	Ad5F35/MKp-E1	A conditionally replicating chimeric adenovirus that has the E1 gene under the control of the human midkine promoter and replaces the fiber knob on Ad5 with that on Ad35	[36]
	Ad/PSCAE/UPII/E1A	An adenovirus with the human UPII promoter PSCAE-regulated E1A gene	[62]
			[63]
	AxdAdB3-F/RGD	An RGD-fiber modified oncolytic adenovirus with E1A and E1B mutations	[57]
	Onco(Ad).RGD-hTERT-TRAIL	An RGD-fiber modified oncolytic adenovirus carrying TNF-related apoptosis-inducing ligand gene and EGFP	[64]
	AdLCY	A hypoxia and Oct4 dual-regulated oncolytic adenovirus	[46]
	Ad5-UPII-E1A	An adenovirus of serotype 5 (Ad5) with the expression of E1A regulated by the uroplakin II (UPII) promoter	[43]
	Ad5/F11p-PSCAE-UPII-E1A	This adenovirus targets BC with its PSCAE and UPII promoters and possesses a chimeric fiber gene that codes for the Ad11p fiber shaft and knob domains, as well as the Ad5 fiber tail domain.	[35]
	XVir-N-31	Also known as Ad-Delo3-RGD, the virus possesses a deletion in the E1A CR3-region, as well as one in the E1B19k gene and the E3 area. There is an RGD motif on the fiber knob.	[44]
	Ad/PSCAE/UPII/E1A-AR	An adenovirus that targets BC and carries E1A-AR controlled by UPII and PSCAE promoters	[47]
Alphavirus	M1	A Getah-like virus with a positive single-strand RNA genome	[39,45]
Coxsackievirus	CVA21	A novel ICAM-1 targeted immunotherapeutic virus	[34]
HSV-1	NV1066	A mutant form of the HSV-1 that has been attenuated, produces green fluorescent protein, and lacks the viral genes ICP0 and ICP4.	[48]
	HF10	A significantly attenuated, replication-capable variant of HSV-1	[42]
	oHSV-1	A HSV-1 mutant that expresses endogenous microR124 and microR143	[65]
HSV-2	FusOn-H2	A HSV-2 mutant that has had the protein kinase domain removed and specifically targets BC cells by activating the Ras signaling system	[40]
Pseudorabies virus	YP2	A mutant of the pseudorabies virus with the HSV-1 thymidine kinase and glycoprotein D genes	[54]
Newcastle disease virus	73-T strain	73-T strain	[53]
	LaSota strain	Recombinant lentogenic NDV LaSota strain	[49]
Vaccinia virus	∆*F4L* VACV	F4L-deleted vaccinia virus	[50]
Reovirus	Reovirus	A double-stranded RNA virus with an icosahedral capsid and no envelope	[38,41]
Vesicular stomatitis virus	AV3	A mutant Delta51M variant	[37]
	AV3	A mutant Delta51M variant encoding a green fluorescent protein	[66]
	VSVd51-hGM-CSF	VSV carring the human GM-CSF transgene	[52]

AR: androgen receptor; BC: bladder cancer; BCG: bacillus Calmette–Guerin; CAR: Coxsackievirus adenovirus receptor; CTLA-4: cytotoxic T lymphocyte-associated antigen 4; CVA21: Coxsackievirus A21; GM-CSF: granulocyte-macrophage colony-stimulating factor: HSV: herpes simplex virus; ICAM-1: intercellular adhesion molecule 1; MK: midkine; MMC: mitomycin C; NDV: Newcastle disease virus; PD-1: programmed cell death protein 1; PSCAE: prostate stem cell antigen enhancer; RGD: Arg-Gly-Asp; UPII: uroplakin II; VSV: vesicular stomatitis virus.

**Table 2:** Summary of clinical trials of oncolytic virus for bladder cancer.

Tumor Type	Virus (Alias)	Parent Virus	Phase	Delivery	Therapy	Status	Results	Trial Identifier
NMIBC	CAVATAK	Adenovirus	I	IVT	monotherapy/combined MMC before TURBT	Completed	Caused noticeable inflammatory alterations in tissue biopsies of NMIBC; no patient reported experiencing any significant toxicities; CR: 1/15	NCT02316171
BCG unresponsive NMIBC	CG0070	Adenovirus	I	IVT	monotherapy	Completed	35 pts included; CR: 48.6%; median duration of CR: 10.4 months. Grade 1–2 bladder toxicities were the most common adverse events observed.	NCT00109655
BCG unresponsive NMIBC	CG0070	Adenovirus	II	IVT	monotherapy	Completed	6-month overall CR: 47%; patients with CIS CR: 50%; acceptable level of toxicity	NCT02365818
BCG unresponsive NMIBC	CG0070	Adenovirus	II	IVT	combining pembrolizumab	Recruiting	NA	NCT04387461
BCG unresponsive NMIBC	CG0070	Adenovirus	III	IVT	monotherapy	Recruiting	NA	NCT04452591
HG NMIBC failed first-line prophylactic intravesical therapy.	OH2	HSV-2	I/II	IVT	monotherapy	Recruiting	NA	NCT05232136
BCG-refractory NMIBC or MIBC undergoes radical cystectomy	MV-NIS	Measles virus	I	IVT	monotherapy as neoadjuvant therapy	Recruiting	NA	NCT03171493
Resectable MIBC	Enadenotucirev	Adenovirus	I	IV	monotherapy as neoadjuvant therapy	Completed	Following IV administration, enadenotucirev was found in the majority of tumor samples and caused significant local CD8+ cell infiltration in 80% of the tumor samples evaluated.	NCT02053220
Advanced BC	Enadenotucirev	Adenovirus	I/II	IV	monotherapy	Completed	Enadenotucirev can be administered in single/repeated cycles with manageable tolerability.	NCT02028442
Advanced BC	CVA21	Coxsackievirus	I	IV	monotherapy/combining pembrolizumab	Completed	Combination therapy was generally well tolerated; median OS: 11.2 mos; ORR: 31% (8/26, 3CR + 5PR); notable increase in PD-L1+ tumor cells: 62% (8/13).	NCT02043665
Advanced BC	CAdVEC	Adenovirus	I	IT	combining chimeric antigen receptor T cells	Recruiting	NA	NCT03740256
Advanced BC	OH2	HSV-2	II	IT	monotherapy	Recruiting	NA	NCT05248789
Advanced BC	PF-07263689	Vaccinia virus	I	IV	monotherapy/combining sasanlimab	Recruiting	NA	NCT05061537
Advanced BC	YSCH-01	Adenovirus	I	IT	monotherapy	Recruiting	NA	NCT05180851

BC: bladder cancer; CIS: carcinoma in situ; CR: complete response; HSV: herpes simplex virus; IT: intratumoral injection; IV: intravenous injection; IVT: intravesical therapy KR: Republic of Korea; MMC: mitomycin C; NA: not available; ORR: objective response rate; OS: overall survival; PD-L1: programmed death-ligand 1; PR: partial response; TURBT: transurethral resection of bladder tumor; UK: United Kingdom; US: United States.

## Data Availability

Not applicable.

## Abbreviations

ACT: adoptive cell therapy; AR: androgen receptor; BC: bladder cancer; BCG: Bacillus Calmette-Guerin; CAR: coxsackievirus adenovirus receptor; CAR-T: Chimeric antigen receptor T cells; CIS: carcinoma in situ; CR: complete response; CTLA-4: cytotoxic T lymphocyte-associated antigen 4; CVA21: Coxsackievirus A21; DAMPs: damage-associated molecular patterns; DCs: dendritic cells; GM-CSF: granulocyte-macrophage colony-stimulating factor; HSV: herpes simplex virus; ICAM-1: intercellular adhesion molecule 1; ICIs: immune checkpoint inhibitors; ICB: immune checkpoint blockade; IT: intratumoral injection; IV: intravenous injection; IVT: intravesical therapy; KR: Korea of Republic; MIBC: muscle-invasive bladder cancer; MK: midkine; MMC: mitomycin C; NA: not available; NDV: Newcastle disease virus; NK: natural killer; NMIBC: non-muscle invasive bladder cancer; ORR: objective response rate; OS: overall survival; OVs: oncolytic viruses; OVT: oncolytic virotherapy; PAMPs: pathogen-associated molecular patterns; PD-1: programmed cell death protein 1; PR: partial response; PrV: Pseudorabies virus; PSCAE: prostate stem cell antigen enhancer; RGD: Arg-Gly-Asp; TAAs: tumor-associated antigens; TCR: cell receptor; TILs: tumor-infiltrating lymphocytes; TK: thymidine kinase; TME: tumor microenvironment; TURBT: transurethral resection of bladder tumor; UK: United Kingdom; UPII: uroplakin II; US: United State; VSV: vesicular stomatitis virus; VV: Vaccinia Virus; ZAP: zinc-finger antiviral protein.
